# Vasculitis-like hemorrhagic retinal angiopathy in Wegener’s granulomatosis

**DOI:** 10.1186/1756-0500-6-364

**Published:** 2013-09-10

**Authors:** Juliane Matlach, Florentina J Freiberg, Ottar Gadeholt, Winfried Göbel

**Affiliations:** 1 Department of Ophthalmology, University of Wuerzburg, Wuerzburg, Germany; 2 Department of Internal Medicine II, University of Wuerzburg, Wuerzburg, Germany

**Keywords:** Granulomatosis with polyangiitis, Wegener’s granulomatosis, Retinal vasculitis, Hemorrhages, Cyclophosphamide

## Abstract

**Background:**

Granulomatosis with polyangiitis, also known as Wegener’s granulomatosis, is a chronic systemic inflammatory disease that can also involve the eyes. We report a case of massive retinal and preretinal hemorrhages with perivascular changes as the initial signs in granulomatosis with polyangiitis (Wegener’s granulomatosis).

**Case presentation:**

A 39-year-old Caucasian male presented with blurred vision in his right eye, myalgia and arthralgia, recurrent nose bleeds and anosmia. Fundus image of his right eye showed massive retinal hemorrhages and vasculitis-like angiopathy, although no fluorescein extravasation was present in fluorescein angiography. Laboratory investigations revealed an inflammation with increased C-reactive protein, elevated erythrocyte sedimentation rate and neutrophil count. Tests for antineutrophil cytoplasmic antibodies (ANCA) were positive for c-ANCA (cytoplasmatic ANCA) and PR3-ANCA (proteinase 3-ANCA). Renal biopsy demonstrated a focal segmental necrotizing glomerulonephritis. Granulomatosis with polyangiitis (Wegener’s granulomatosis) was diagnosed and a combined systemic therapy of cyclophosphamide and corticosteroids was initiated. During 3 months of follow-up, complete resorption of retinal hemorrhages was seen and general complaints as well as visual acuity improved during therapy.

**Conclusion:**

Vasculitis-like retinal changes can occur in Wegener’s granulomatosis. Despite massive retinal and preretinal hemorrhages that cause visual impairment, immunosuppressive therapy can improve ocular symptoms.

## Background

Granulomatosis with polyangiitis (Wegener’s granulomatosis) is a chronic systemic inflammatory disease. The pathophysiological correlate of the disease is a small-vessel vasculitis with necrotizing granulomatous lesions of the upper and lower respiratory tract, the kidneys and other organs. Clinical signs and symptoms are nonspecific and can therefore resemble other vasculitic disorders that affect small- and medium-sized vessels. Ophthalmic manifestations occur in up to 60% of patients and may be the initial clinical signs. Wegener’s granulomatosis can affect any part of the eye and may cause conjunctivitis, episcleritis and scleritis, keratitis, uveitis, retinal vasculitis, and involvement of the orbit, eyelid and nasolacrimal drainage system [1-3].

We report a case of Wegener’s granulomatosis with massive retinal hemorrhages as the initial presenting sign which resolved with immunosuppressive therapy.

## Case presentation

**Figure 1:**
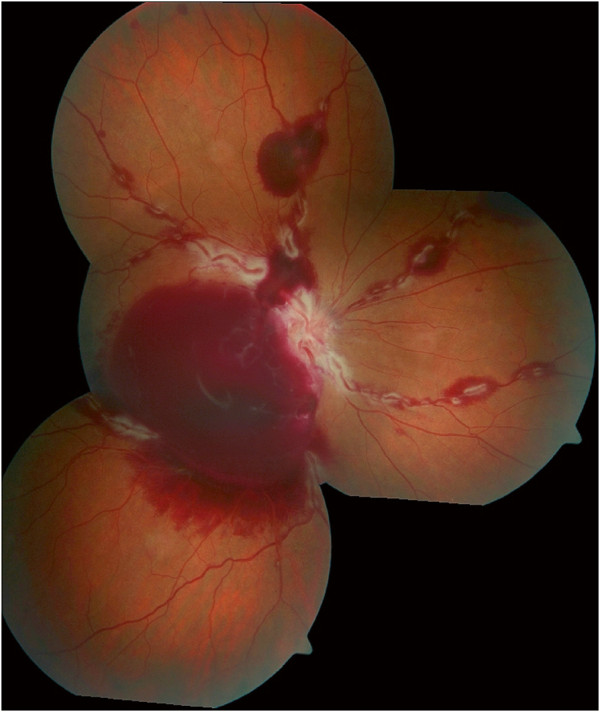
**Right eye fundus image at first presentation.** Dilatation of retinal veins, retinal and preretinal retrohyaloidal hemorrhages and segmental perivascular changes.

**Figure 2:**
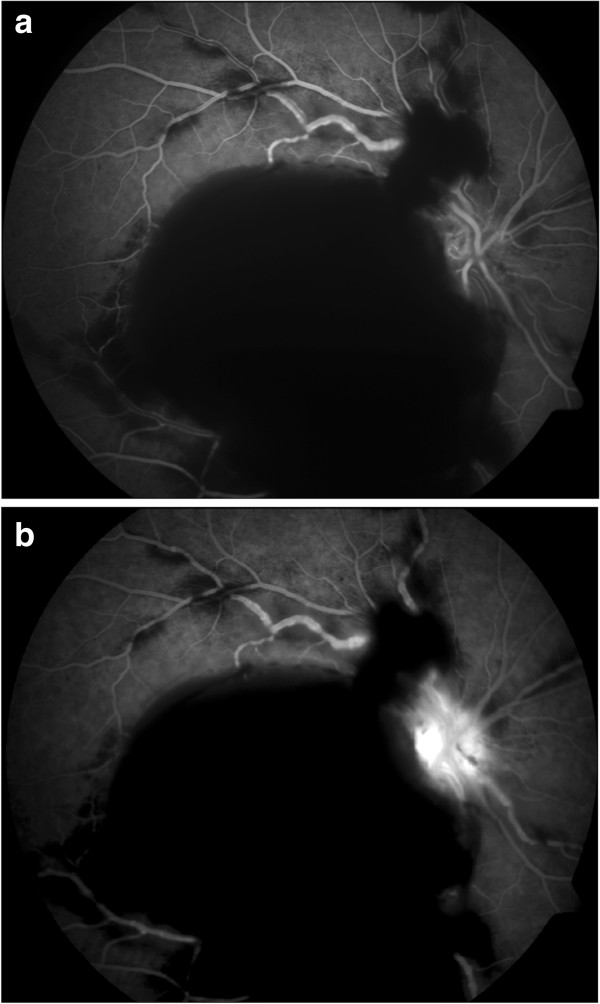
**Fluorescein angiography of the right eye at first presentation.** Blocked fluorescence as a result of massive retinal hemorrhages, engorgement of retinal veins with staining of the vessel wall (**a**, arteriovenous phase, 0:21 min.). No fluorescein extravasation in the late phase. Fluorescein leakage of the optic disc due to ischemia (**b**, 4:13 min).

A 39-year-old Caucasian male presented with decreased visual acuity of 20/400 in his right eye since the day before. Slit lamp biomicroscopy of the anterior segment OD confirmed a slightly injected conjunctiva. Fundus examination of his right eye showed multiple retinal and preretinal hemorrhages, dilatation of retinal veins and perivascular changes (Figure 1). Fluorescein angiography revealed engorgement of retinal veins and staining of the vessel wall without fluorescein extravasation in the late phases (Figure 2a, b).

Furthermore, he complained of having conjunctivitis in his left eye for 6 weeks. Visual acuity in his left eye was 20/20. Slit lamp biomicroscopy of the anterior segment showed a hyperemia of the conjunctiva, while fundus examination was unremarkable.

At that time, he reported a 4-month history of generalized steroid-responsive myalgias and finger joint pain and a 4-year history of chronic sinusitis and frequent nose bleeds.

Routine laboratory investigations and special laboratory studies for infectious and autoimmune diseases, as well as otolaryngologic and internistic examination were performed.

### Laboratory diagnostics

Routine laboratory testing revealed an increase in neutrophil count of 8.10 x 10^9^/L (normal range 1.8-7.2 × 10^9^/L), an elevated erythrocyte sedimentation rate (ESR) of 41 mm in the first hour (normal range 0–15 mm/hour) and a C-reactive protein (CRP) of 76.5 mg/L (normal range 0–5 mg/L). Urinary tests and microscopic examination showed hematuria with dysmorphic erythrocytes (red blood cells 44/μL; normal range 25/μL) and proteinuria (albumin 434 mg/L; normal range 30 mg/L). Serological testing excluded recent infectious diseases.

Additional tests for antineutrophil cytoplasmic antibodies (ANCA) were performed and showed a positive c-ANCA (cytoplasmatic ANCA) titer of 1:640 (negative <1:40) and a proteinase 3-ANCA (PR3-ANCA) value of >100 IU/mL (normal 3.5 IU/mL). Anti-myeloperoxidase antibodies (MPO-ANCA) were within the normal range (< 9 IU/ml).

#### Otolaryngologic examination

Erosive changes and irregular mucosal thickening were present in the nasal turbinates. Biopsy of mucosa was nonspecific. Loss of mucosa had caused dryness, crusting, epistaxis and anosmia. Computed tomography of the paranasal sinuses showed irregular mucosal thickening, but no destruction of the nasal septum or sinus walls.

#### Renal biopsy findings

On histopathological examination, the renal biopsy demonstrated a focal segmental necrotizing glomerulonephritis.

#### Therapy

According to the American College of Rheumatology (ACR) criteria, granulomatosis with polyangiitis (Wegener’s granulomatosis) was diagnosed [4]. The patient received intravenous corticosteroids (250 mg prednisolone per day for 3 days tapering over weeks; maintenance dose: 7.5 mg) and cyclophosphamide 750 mg/m^2^ (accumulated dose: 11110 mg) for 7 pulses. A good response with remission of symptoms and reduced c-ANCA and PR3-ANCA values was noted. After cyclophosphamide and corticosteroids had induced remission, subcutaneous methotrexate (25 mg) was given once weekly to maintain remission. The renal function, initially slightly impaired (GFR 72 ml/min/m^2^) was normalized. The proteinuria improved from a maximum of 2210 mg/l (albumin/creatinine [a/c] ratio 1177 mg/g) to currently 351 mg/l a/c 238 mg/g.

**Figure 3:**
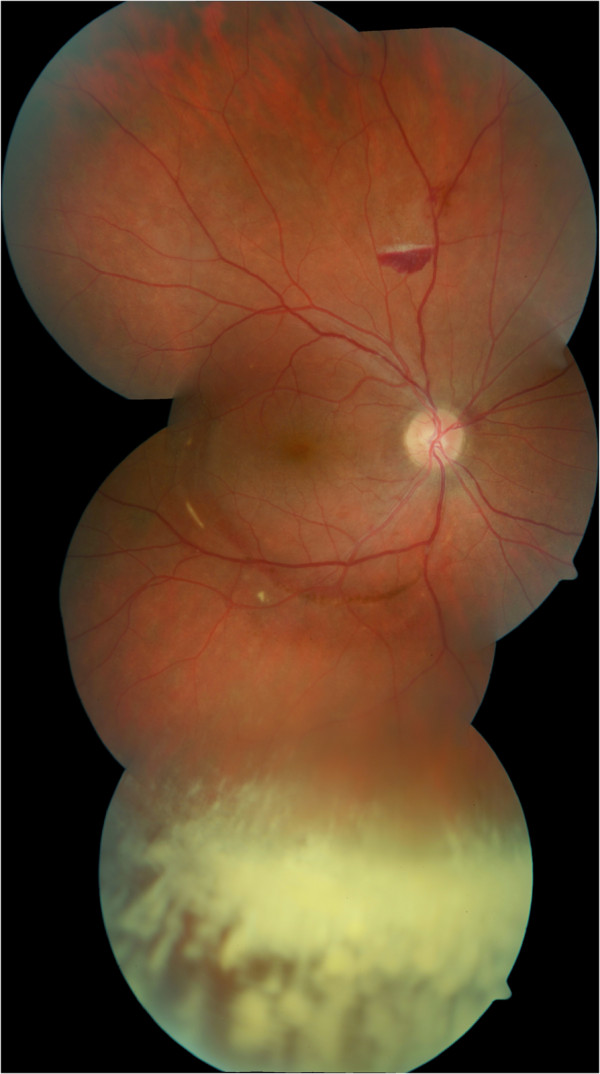
**Right eye fundus image 3 months after initial presentation.** Complete resorption of central retinal hemorrhages and remnants of vitreous hemorrhage inferiorly.

Complete resorption of central retinal hemorrhages was seen 3 months after initial presentation and visual acuity improved to 20/20 in his right eye (Figure 3). There was no evidence of afferent pupillary defect or optic nerve atrophy.

## Discussion

Granulomatosis with polyangiitis (Wegener’s granulomatosis) is a systemic inflammatory disease characterized by granulomatous vasculitis that frequently involves the upper respiratory tract, lungs, kidneys and eyes. Retinal vasculitis with retinal artery or vein occlusion, chorioretinitis, macular edema, exudative retinal detachment and serious retinal necrosis are rare complications and are usually associated with poor visual prognosis [2,5-7].

We found clinical signs of a vasculitis-like retinal angiopathy without fluorescein extravasation in angiography. This is in line with case reports of retinal vein occlusion in Wegener’s granulomatosis without clinical and angiographic signs of vasculitis [5-7]. Contrary to those reports, the perivascular tissue of retinal veins was considerably affected in our case.

A combined regime of corticosteroids and cyclophosphamide has been demonstrated to achieve remission in the majority of patients and is recommended for patients with a more severe form. Surgical interventions to remove retinal and preretinal hemorrhages should be limited to those patients who do not respond to medical therapy. In our case, even massive retinal and preretinal hemorrhages resolved within 3 months without surgery.

## Conclusion

This case report demonstrates that massive retinal and preretinal hemorrhages in Wegener’s granulomatosis can resolve under immunosuppressive therapy. Despite the lack of acute signs of vasculitis, particularly fluorescein extravasation in angiography, severe vasculitis-like retinal changes can occur leading to diagnosis of Wegener’s granulomatosis.

## Consent

Written informed consent was obtained from the patient for publication of this Case report and any accompanying images. A copy of the written consent is available for review by the Editor of this journal.

## Abbreviations

ANCA: Antineutrophil cytoplasmic antibodies; PR3-ANCA: Proteinase 3-ANCA; c-ANCA: Cytoplasmatic ANCA; OD: Oculus Dexter; ESR: Erythrocyte sedimentation rate; CRP: C-reactive protein; MPO-ANCA: anti-myeloperoxidase antibodies; ACR: American College of Rheumatology; GFR: Glomerular filtration rate; a/c: albumin/creatinine.

## Competing interests

All authors declare that they have no competing interests.

## Authors’ contributions

JM has collected the data of the case report and drafted the manuscript. FJF and OG critically revised the manuscript. WG was involved in drafting the manuscript and has given final approval of the version to be published.
